# Balancing patient safety and sustainable healthcare for orthopaedic device utilization in resource-limited countries: insights from the REBOOT Study

**DOI:** 10.1186/s13037-026-00475-1

**Published:** 2026-04-17

**Authors:** REBOOT Study Group, Emmanuel O. Oladeji, Olorunnisola Olatide, Oluwafisayo Awi, Ridwanullah O. Abdullateef, Abdullahi Ringim, Abdulahi Zubair, Imobhio G. Okhifun, Abdulquddus Ajibade, Noah B. Oyedokun, Oluwatobi Olayode, Patrick Okonkwo, Bismarck C. Iwueke, Japheth O. Olaku, Saidu I. Alhaji, Suleiman T. Olorukooba, Toluwani O. Orungbeja, Michael K. Nzeako, Emamizo U. Ojo-maliki, Hadayat Ullah, Adedamola Olaniyi, Tochukwu Enemuo, Oghofori Obakponovwe, Samuel O. Ogunlade, Abdul K. Bah, Abidemi M. Ayodele, Abiola M. Fawale, Abrar U. Haq, Adeola S. Adeniran, Kayode A. Adesunkanmi, Adewura R. Kazeem, Adeyemi D. Ogunoye, Ahmad Khan, Aiman Khan, Ajibade M. Oyegade, Akinlolu M. Adewolu, Akintunde Oyelami, Akinyemi S. Akinpelumi, Alex M. Kihunyu, Anayochukwu J. Oke, Aniebiet Ubaha, Babatunde A. Osundina, Bernard Hammond, Bode L. Afeniforo, Bukar Bunu, Busari B. Animasaun, Charles K. Aisudo, Chioma M. Ajuyah, Cyprian C. Nganwuchu, Dahiru Sani, Eveshoyan E. Daniels, David K. Kokor, Dung D. Chong, Emeka C. Elumelu, Fatiat O. Ayoade, Franklin A. Achumba, Friday J. Leba, Gershom E. Igwe, Hammed O. Alabi, Hardy Elembwe, Hari E. Akachuku, Ibrahim Sabo, Idowu O. Alaba, Igbokwe C. Okwuchukwu, Isaac M. Ahorklo, Jemiludeen O. MorhasonBello, Jesse Tanko, John A. James, Kabir K. Bello, Killian T. Ninnang, Kingsley I. Egwuonwu, Kolawole S. Ayo-Oladapo, Adetutu O. Lebi, Margaret O. Popoola, Mohammed A. Aliyu, Muhammed O. Bashiru, Musiliu A. Oladosu, Nasiru Abba, Nnaemeka A. Alor, Nura M. Aliyu, Sakirudeen A. Olamide, Olusegun O. Olanipekun, Olasode I. Akinmokun, Olusegun E. Ayediran, Oluwadayo A. Magbagbeola, Oluwafeyikemi O. Olabisi, Oluwajimi Gbotoso, Oluwasegun A. Aremu, Oluwaseun A. Amodu, Oluwaseyi K. Idowu, Oluwatoyin E. Olasehinde, Oyedele S. Olaoye, Prince C. Okolo, Qazeem B. Adesola, Rahman A. Afolabi, Reuben M. Gibil, Rich-Hope O. Abiye-Whyte, Ridhwanullah A. Salawu, Ridwan O. Maleeq, Ronald A. Williams, Saddam Hussain, Sadiq Tijani, Sakirudeen A. Olamide, Samuel B. Agaja, Samuel T. Oladejo, SatiSamson Bawa, Segun J. Oni, Selim S. Oriloye, Senyo Gudugbe, Sherif T. Maruf, Solomon J. Fadamijo, Taofikat Ikotun, Tathiya N. Fakuta, Tavershima M. Tsavzua, Temidayo J. Alarapon, Temitope J. Oyadiran, Timothy K. Olagbe, Tobiloba E. Oyeyemi, Tolulope O. Ogunrewo, Udit Agrawal, Umar Mohammed, Uno O. Okpo, Vernon Ipomai, Victor Akinkuolie, Yashim I. Ignatius, Yau Musa, Yitmwa Ngwan, Yusuf O. Zakariyau

**Affiliations:** Trauma and Orthopaedics Department, Surgery Interest Group of Africa, Km 43, Lekki-Epe Expressway, Abijo, Ibeju-Lekki, Lagos State Nigeria

**Keywords:** Orthopaedic devices, Sterile reprocessing, Cost containment, Sustainability in surgery, Resource-limited settings

## Abstract

**Background:**

Access to orthopaedic care in low- and middle-income countries is limited by device scarcity and high costs, despite the significantly greater burden of trauma. The reuse of reprocessed implants and equipment has emerged as a pragmatic solution, but safety assurance and governance remain inconsistent. We described current reuse practices in low- and middle-income countries, evaluated safety risks and mitigation strategies to balance equity and sustainability with patient protection, and highlighted opportunities to reduce healthcare costs.

**Methods:**

We conducted a multinational, cross-sectional survey of orthopaedic clinicians in hospitals in low- or middle-income countries between June 2024 and February 2025. Primary outcomes were device-specific reuse prevalence and safety governance; secondary outcomes were reuse drivers and risks.

**Results:**

A total of 113 respondents from 36 hospitals in seven low- and middle-income countries participated. The majority (84%) worked in university-based hospitals or postgraduate training centres, and most (78%) were orthopaedic and spine consultants or residents. External fixator components were most frequently reused (80%), with implantable pins or wires reused by 67%; 51% reported using reprocessed implantable devices at least weekly. Only 35% knew the exact source of reused implants; devices mostly originated from explants or de-sterilised but unimplanted stock. 55% used reprocessed implants without any quality testing or recertification, and 34% were unsure; just 8% reported formal re-evaluation. Ninety-eight per cent were unaware of any applicable guidelines. The reuse of reprocessed devices did not differ significantly by hospital affiliation, hospital resourcing, level of care, or respondent’s years of orthopaedic experience. Perceived benefits were affordability and access, while reported concerns included device failure, ethical/litigation issues, and patient disapproval—all amplified by variable reprocessing standards and poor traceability.

**Conclusions:**

The use of reprocessed orthopaedic biomaterials is common in low- and middle-income countries. External fixator components are the most reprocessed implants, with cost and availability as the primary drivers. Unclear sourcing, weak quality assurance, and absent guidance create avoidable safety risks. Safer practice requires context-specific standards for sourcing/reprocessing, validated decontamination and sterilisation with full traceability, risk-stratified reuse, mandatory quality testing/recertification of reprocessed implants, informed consent that addresses reuse, and regular audit of practice compliance.

**Supplementary Information:**

The online version contains supplementary material available at 10.1186/s13037-026-00475-1.

## Introduction

The challenges facing orthopaedic care, including implants and devices procurement, in low and middle-income countries are considerable [1, 2], matching 90% of the global burden of trauma deaths coming from these geographical areas [1, 3, 4]. Despite the disproportionately high trauma burden in low- and middle-income countries along with the correspondingly high rate of preventable disability-adjusted life years from injuries [5], access to surgical services remains limited [6–8].

The scarcity of orthopaedic equipment and fixation implants constrains care [9–11]. At the same time, high out-of-pocket surgical costs—amid limited insurance coverage—undermine universal health-coverage goals, leaving many patients in low- and middle-income countries unable to access needed care [2, 12–14]. The prevailing reality in these settings is driving the use of reprocessed orthopaedic devices [15]. Yet, evidence is sparse on what is reused, how safety is assured, and the other drivers of uptake in low- and middle-income countries [12, 16–19].

The reuse of designated orthopaedic surgical instruments is a standard practice. These instruments are designed for repeated cycles and must undergo validated cleaning, disinfection, sterilisation, and storage after each use, in compliance with relevant manufacturer and regulatory best practices [20, 21]. On the other hand, opened but unused implants, typically generated by opening the incorrect implant (wrong size or side), a change in the operative plan, opening incompatible implants, or inaccurate measurements, may be reprocessed with documented, validated procedures [22]. Implants that were previously patient-contacted require stricter controls with full traceability.

To bridge the gap in the scope and practice of safe biomaterial reuse and to drive further research, we conducted the Reprocessed Biomaterials Use in Orthopaedic Surgery Study (REBOOT Study). We described the practices surrounding the reprocessing of orthopaedic implants and devices in contemporary orthopaedic practice in low- and middle-income countries. We highlighted opportunities for healthcare cost savings and for greening operating theatres, and the crucial need to find a desirable balance between maximising patients’ safety, minimising waste production, and reducing healthcare costs.

## Methods

The REBOOT Study employed a multinational, cross-sectional, web-based survey of orthopaedic clinicians, including consultants or attendings, trainees or residents, and career-grade specialists, who were actively practising in hospitals within low- and middle-income countries [23, 24]. We hypothesised that reuse of reprocessed orthopaedic implants and devices is prevalent in these settings and that safety governance measures, including traceability, quality testing or recertification, and written guidance, are inconsistently implemented.

The primary outcomes were the prevalence of reuse by device category and the presence of safety governance, including knowledge of source or traceability, quality testing or recertification, and awareness of written guidance. Secondary outcomes encompassed the drivers of reuse, perceived risks, frequency of reuse, sourcing and reprocessing locations, and associations between reuse practices and hospital affiliation, hospital resourcing, level of care, and years of experience.

Respondents were recruited from hospitals in low- and middle-income countries. Most sites were tertiary or university-affiliated centres, with a minority representing secondary-level hospitals. Eligible participants were clinically active orthopaedic clinicians working in low- and middle-income country hospitals, with at least two years of post-internship orthopaedic experience, able to complete the survey in English or French, and who provided informed electronic consent. Exclusion criteria included non-clinical roles, less than two years of post-internship orthopaedic experience, or incomplete responses. Ethical approval was not required as the participation process did not involve patients and was voluntary.

A 28-item survey instrument, available in English and French, was developed based on prior literature, pilot-tested, and disseminated through professional networks using a snowball sampling approach. The survey collected data on respondent demographics, hospital characteristics, types of devices reused, sourcing and reprocessing pathways, quality testing or recertification, guidance or policies, frequency of reuse, perceived drivers and risks of reuse, and experiences with complications and operative time. Data collection occurred between 1 June 2024 and 28 February 2025. All responses were anonymised.

Statistical analyses were conducted using Python (v3.10.12). Data were summarised descriptively, and categorical variables were analysed using Fisher’s exact tests. *P* values < 0.05 were considered statistically significant.

## Results

### Respondents and hospital profile

**Fig. 1:**
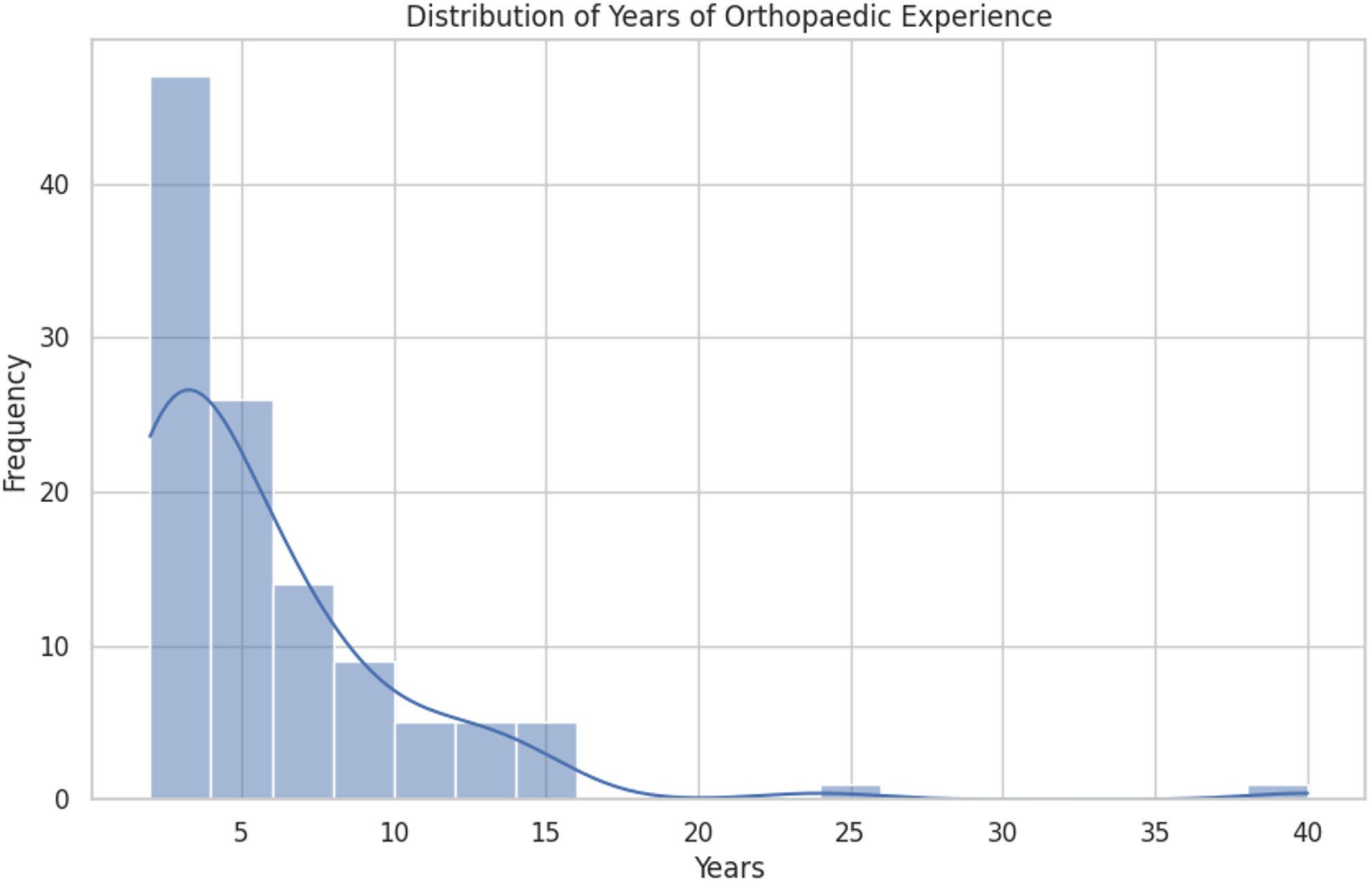
Distribution of years of experience of respondents

One hundred and thirteen complete responses were received. Respondents have varying levels of postgraduate orthopaedic experience and work in 26 hospitals across seven low- or middle-income countries. Approximately one-quarter (27) were orthopaedic consultants; 62 were orthopaedic residents; the remainder were medical officers (career-grade specialists) who practice under the supervision of orthopaedic consultants. The majority of the consultants were general orthopaedic and trauma surgeons, while only a few were subspecialists in complex trauma, arthroplasty, arthroscopy, spine surgery, or paediatric orthopaedics. All respondents have practised orthopaedics for 2 to 40 years, with an average of 4 years; almost half (47%) had practised for at least 5 years. (Fig. 1).

**Fig. 2:**
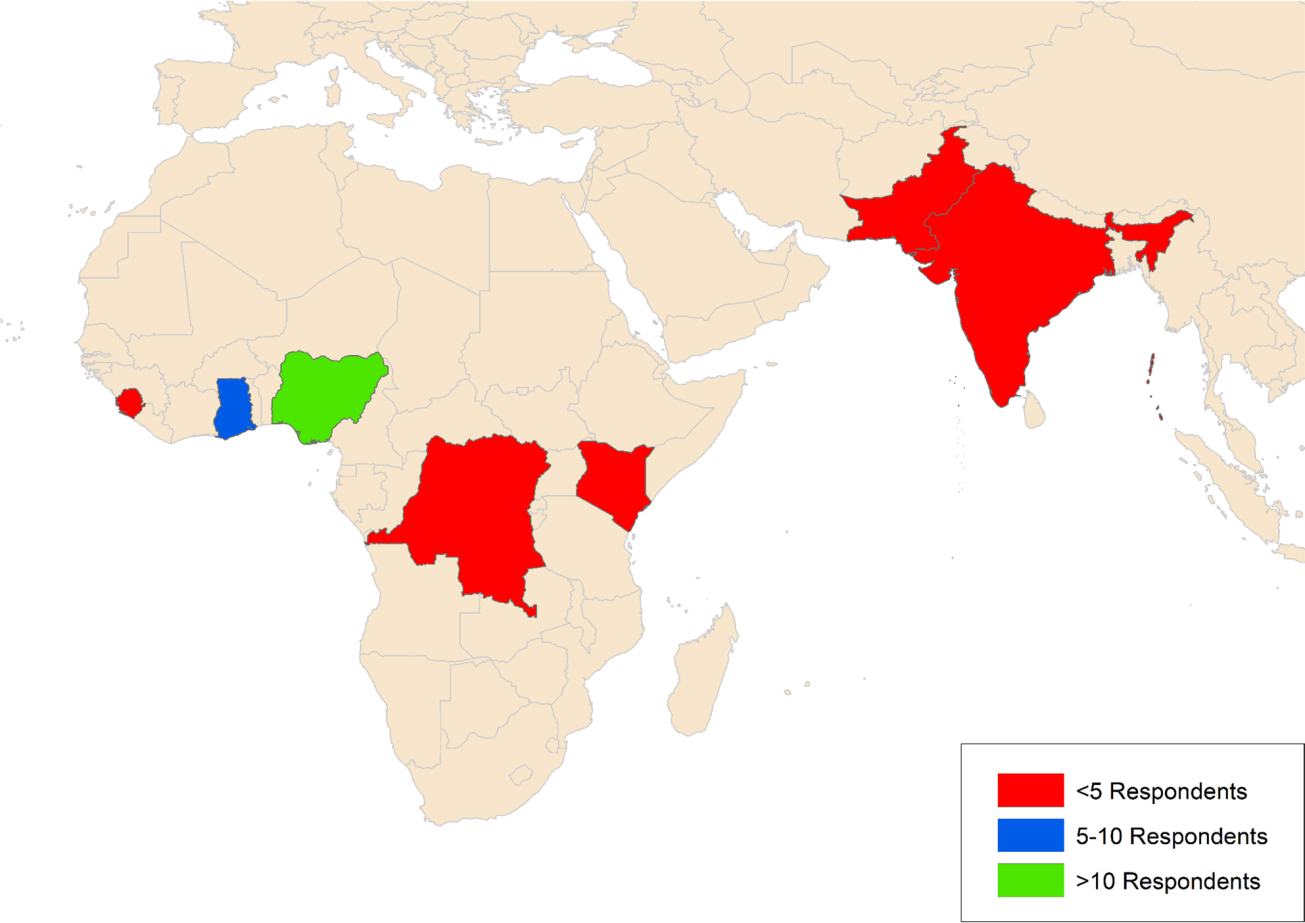
The distribution of respondents and country of practice

26 out of the 36 hospitals are in Nigeria, three each in Ghana and Pakistan, and one each in Kenya, the People’s Democratic Republic of Congo, India, and Sierra Leone. The distribution of respondents is shown in Fig. 2.

Ninety-five (84%) of the respondents practised in tertiary care centres, and the remainder practice in secondary care centres. Seventy-nine (70%) practised in urban areas, while 29 and five practised in suburban and rural areas, respectively.

Thirty-three of the hospitals were funded by government revenue, whereas the other three were privately funded and profit-oriented. Twenty-nine of the hospitals were either teaching hospitals affiliated to a university or stand-alone postgraduate training centres; the rest were general and private hospitals.

### Reprocessed devices usage

**Fig. 3:**
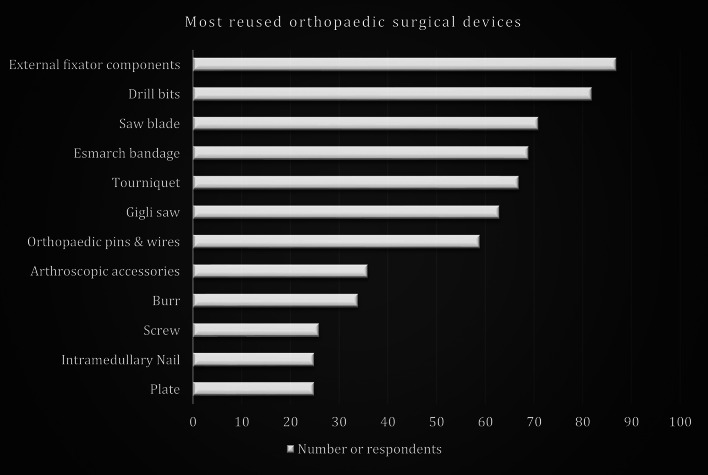
Reprocessed implants and devices used by respondents

The most used reprocessed implants and devices by respondents are shown in Fig. 3. They included external fixation components such as the linear rail system and circular frames, and implantable devices such as plates, screws, intramedullary nails, wires, and pins. Fifty-one per cent of respondents use reprocessed implants and devices at least once a week. These devices include drill bits, Gigli saw, Esmarch bandage, and arthroscopic gadget accessories. General orthopaedic surgeons use these reprocessed implants and devices more often (83%) than subspecialists. Fifty-seven per cent of respondents reuse reprocessed implants and devices multiple times until visibly damaged, compared with 87% who reuse consumables such as gowns and drapes similarly. We found no statistically significant difference between reprocessed device usage and hospital resourcing, level of care, or years of orthopaedic experience.

### Sources and reprocessing of implants, devices and consumables

Thirty-five per cent of respondents were aware of the source of the reprocessed implants. Sources included implants removed from other patients electively or discarded during a procedure after being de-sterilised (but not implanted) (Table 1). Two-thirds of the respondents reprocessed devices in the same hospital where they practice or at a different hospital. Thirteen of them use a private reprocessing unit or the device’s original manufacturer or vendor, whereas the remainder (20) are unsure where the devices are reprocessed.

A notable unpublished institutional experience is that of a tertiary national orthopaedic specialist centre in Nigeria, where used hexapods imported from overseas are routinely reused for patients due to the high cost of new ones. The hexapods undergo the institution’s standardised moist-heat sterilisation procedure utilising an autoclave.

Fifty-five per cent of respondents use reprocessed implants and devices without any quality testing or recertification; 34% are not sure of the quality testing status of devices they reuse, while 8% ensure the quality and recertification of devices they reuse, although the process of quality testing and recertification was not provided (Table 1).

**Table 1:** Practices regarding the use of reprocessed surgical fixation devices

Practice	*N* (%)
*Where the devices are reprocessed*	
The same or different hospital	72 (68.5)
Unsure	20 (19.1)
A private reprocessing unit	9 (8.6)
The original device manufacturer	4 (3.8)
*Quality testing or re-evaluation/recertification of devices*	
Yes	7 (8)
No	49 (55)
Sometimes	3 (3)
Unsure	30 (34)
*Awareness of where the device is sourced*	
Yes	31 (35)
No	24 (27)
Sometimes	33 (38)
*Sources of reprocessed implants*	
Explanted and discarded during a procedure	77 (85.6)
Implant de-sterilised during a procedure	46 (51.1)
Implant removed at patient’s request	44 (49)
Implant removed on patient’s demise	9 (10)

### Reasons and motivations for using reprocessed implants and devices

Affordability and availability were the most cited reasons for reusing reprocessed orthopaedic devices (Table 2). All respondents use reprocessed implants and devices because they are cost-effective, saving costs for the patient, the surgeon, and/or the hospital. Seventeen per cent of the respondents believe that reusing equipment and consumables is environmentally friendly, in addition to their cost-saving advantage. More training opportunities for orthopaedic residents, due to the affordability of reprocessed devices for patients or the donation of explanted implants to indigent patients, are another motivation for reuse.

**Table 2:** Reasons and motivations for using reprocessed implants and devices

Attribute	*N* (%)
*Reasons for using reprocessed devices*	
Cost savings for the patient/hospital	74 (84)
High cost of newly manufactured alternative	58 (66)
Problems with the availability of new device	50 (57)
More income generation for the hospital	37 (42.5)
Environmentally friendly	15 (17)
More income generation for the surgeon	9 (10.2)
*Reasons for not using reprocessed devices*	
Lack of knowledge about reprocessing logistics	14 (67)
Ethical concerns	13 (62)
Patient disapproval	12 (57)
Concern about implant failure	11 (52.4)
Litigation concerns	10 (47.6)
Local policy	6 (28.6)
Lack of infrastructure for reprocessing	4 (19)
*Experience with using reprocessed devices*	
Cost savings for the patient	84 (92)
Cost savings for the hospital	47 (51.6)
Easier access when compared with new devices	34 (37.4)
Does not affect complication rate	33 (36.3)
Does not affect surgery time	28 (31)
Prolongs surgery	18 (20)
Higher complication rates	17 (18.7)
No significant cost savings	2 (2.2)

Aside from cost savings for patients and care providers, most respondents also reported greater availability than for newly manufactured devices. However, the respondents’ experiences with complications and operation time were mixed. While some have experienced higher complication rates and prolonged operating time, others have had the opposite experience. However, objective data to substantiate these experiences were lacking.

Ninety-eight per cent of respondents reported not knowing any relevant practice guidelines, suggesting wide variability in practice across centres. Seventeen per cent of respondents expressed reservations about using reprocessed surgical devices. Ethical considerations, patient disapproval, lack of knowledge about reprocessing logistics, and concerns about implant failure attributed to defective devices encountered after multiple reuses were the most frequently cited reasons (Table 2).

## Discussion

In orthopaedic surgery, devices such as screws, plates, rods, and prostheses are essential for procedures including fracture fixation, joint replacement, and spinal surgery. In low-resource settings, high trauma volumes, infrastructure gaps, and out-of-pocket care create strong incentives to expand access through lower-cost device strategies while maintaining operational efficiency [25]. Accordingly, many hospitals reprocess these devices to reduce costs while providing patient care [26]. This is the first study to explore the scope of reprocessed orthopaedic surgical devices in low-resource settings. The findings show that reprocessing biomaterials in orthopaedic and trauma surgery is a common practice in low- and middle-income countries, with Nigeria representing a prototype of such a setting.

The reuse of reprocessed non-implantable components of external fixators has been previously described and well-researched [18, 27, 28]. Our study reinforced the widespread utilisation of these devices, with the external fixator emerging as the most reprocessed implant. However, we found that implantable components of external fixator devices are commonly reprocessed in low- and middle-income countries, along with plates for osteosynthesis, screws, intramedullary devices, and Kirschner wires.

Evidence supports that non-implantable components (bars, clamps) of external fixators can be safely reused under validated programmes, with a randomised trial showing no increase in pin-tract infection, fixation loss, or loosening when refurbished parts were used [29]; their principal hazard is mechanical wear, mitigated by inspection criteria and reuse limits. Pins and wires, by contrast, traverse skin and contact bone, creating a colonisation conduit and higher biological risk. Reuse of other implantable devices (plates, screws, nails, K-wires) and tissue-cutting instruments (drill bits, saw blades) is associated with similar risks and warrants stricter device-level quality assurance, traceability, and reuse criteria.

Cost and availability are the primary drivers of reprocessed device use. In low- and middle-income countries, heavy reliance on imported medical devices inflates procurement costs, while high out-of-pocket spending further limits access [13]; reprocessing is therefore adopted as a cost-containment adaptation, consistent with economic rationales reported in higher-income settings [27, 30, 31]. Limited access to suitable implants (compelling off-label use of available ones) was exemplified in a recent case report, which highlighted a cheaper solution of intramedullary nail fixation for a long-bone fracture [11].

Respondents with a broad range of orthopaedic experience (2–40 years), who are mostly practising in not-for-profit postgraduate training centres, report primarily reusing devices to expand affordable access, potentially narrowing inequities and increasing operative caseloads to support the training of orthopaedic residents. Surgeons generally view this as acceptable; reservations were uncommon and centred on ethics, potential patient disapproval, the risk of implant failure, and occasional defects after multiple reuses. These concerns underscore the absence of standardised quality testing and recertification protocols and reinforce the need for a framework to ensure safe reuse. *This practice may persist and perhaps increase due to the general lack of robust health insurance coverage in these low-resource settings*,* with no sign of a paradigm shift in the near future* [13].

Respondents who avoided reprocessed implants chiefly cited infection and failure risk. Although a randomised trial of reused non-implantable external fixator components found no increase in pin-tract infection or implant failure [29], evidence on the reuse of implantable components is lacking. Given the higher orthopaedic surgical site infection rates in low- and middle-income countries [32, 33], this research gap warrants study, alongside the potential harms of repeated sterilisation—corrosion, material degradation, and loss of mechanical integrity—particularly for cutting tools and implants [34].

Many respondents reported reusing implants without knowing their source or reprocessing method. Clear traceability would allow surgeons to judge safety and reassure patients. One centre’s autoclave-based moist sterilisation mirrors Danesi et al.’s protocol, which adds ultrasonic and manual decontamination with post-cleaning inspection to confirm fitness for reuse [35].

This study has some shortcomings and limitations. Firstly, the insights derived from this study are those of orthopaedic practitioners alone. Some of the insights may therefore be one-sided and cannot be assumed to apply to the patients. Further study is necessary to determine patients’ perceptions of the use and benefits of reprocessed devices. Such further study would also shed more light on the ethical issues surrounding the use of reprocessed implants and devices, especially in the areas of autonomy and non-maleficence. Additionally, most respondents are from a single country; hence, the practices described may not reflect general practices in other countries that are underrepresented among the respondents. Finally, there is a potential for recall bias whereby respondents may inaccurately report past events.

## Conclusion

In our cohort, reuse was most common for external fixator components (80%), followed by drill bits (73%) and saw blades (63%), driven primarily by patient/hospital cost savings, high new-device prices, and limited availability. As the socio-economic and healthcare indices in these geographical areas continue to worsen despite increasing trauma and orthopaedic burdens, the use of reprocessed surgical fixation devices is likely to continue to rise. This solution, while imperfect, ensures that trauma and orthopaedic care are within the reach of more patients and remains a viable strategy for achieving Sustainable Development Goals by making care affordable in low-resource settings.

To limit the risks associated with implant reuse, we recommend multidisciplinary collaboration to develop contextually appropriate regulatory standards and guidelines, and to implement enforcement mechanisms for sourcing, traceability, reprocessing and quality assurance, and using these devices in low- and middle-income countries, to ensure consistent practices and patient safety. Further studies are needed to assess infection rates associated with implantable devices and to determine patients’ experience and acceptance of this practice.

## Supplementary Information

Below is the link to the electronic supplementary material.

Supplementary Material 1

## Data Availability

The datasets used and/or analysed during the current study are available from the corresponding author upon reasonable request.
