# Supplementary material for: Balancing patient safety and sustainable healthcare for orthopaedic device utilization in resource-limited countries: insights from the REBOOT Study
Source: Patient Saf Surg. 2026 Apr 17;20:15. doi: 10.1186/s13037-026-00475-1 (PMC13088594; doi:10.1186/s13037-026-00475-1)
Supplement: Supplementary file 1 — Supplementary Material 1 [file 13037_2026_475_MOESM1_ESM.docx]

**Collaborating authors**

Abdul K Bah^1^, Abidemi M Ayodele^2^, Abiola M Fawale^3^, Abrar U Haq^4^, Adeola S Adeniran^5^, Kayode A Adesunkanmi^6^, Adewura R Kazeem^7^, Adeyemi D Ogunoye^8^, Bode L Afeniforo^6^, Ahmad Khan^9^, Aiman Khan^10^, Ajibade M Oyegade^11^, Akinlolu M Adewolu^5^, Akintunde Oyelami^12^, Akinyemi S Akinpelumi^13^, Alex M Kihunyu^14^, Nura M Aliyu^15^, Anayochukwu J Oke^16^, Aniebiet Ubaha^12^, Babatunde A Osundina^3^, Bernard Hammond^17^, Bismarck C Iwueke^18^, Bukar Bunu^19^, Busari B Animasaun^3^, Charles K Aisudo^7^, Chioma M Ajuyah^2^, Cyprian C Nganwuchu^16^, Dahiru Sani^20^, Eveshoyan E Daniels^12^, David K Kokor^17^, Dung D Chong^18^, Emamizo U Ojo-maliki^13^, Emeka C Elumelu^2^, Fatiat O Ayoade^5^, Franklin A Achumba^2^, Friday J Leba^20^, Gershom E Igwe^3^ Hadayat Ullah^9^ Hammed O Alabi^21^, Hardy Elembwe^22^, Hari E Akachuku^2^, Ibrahim Sabo^15^, Idowu O Alaba^6^, Igbokwe C Okwuchukwu^18^, Isaac M Ahorklo^17^, Japheth O Olaku^18^, Jemiludeen O MorhasonBello^2^, Jesse Tanko^2^, John A James^23^, Kabir K Bello^20^, Killian T Ninnang^24^, Kingsley I Egwuonwu^7^, Kolawole S Ayo-Oladapo^3^, Adetutu O Lebi^25^, Margaret O Popoola^20^, Michael K Nzeako^25^, Mohammed A Aliyu^20^, Muhammed O Bashiru^6^, Musiliu A Oladosu^6^, Nasiru Abba^15^, Nnaemeka A Alor^1^^6^, Sakirudeen A Olamide^13^, Olusegun O Olanipekun^26^, Olasode I Akinmokun^27^, Olusegun E Ayediran^25^, Oluwadayo A Magbagbeola^7^, Oluwafeyikemi O Olabisi^25^, Oluwafisayo F Awi^2^, Oluwajimi Gbotoso^27^, Oluwasegun A Aremu^2^, Oluwaseun A Amodu^3^, Oluwaseyi K Idowu^3^, Oluwatoyin E Olasehinde^11^, Oyedele S Olaoye^2^, Prince C Okolo^16^, Qazeem B Adesola^3^, Rahman A Afolabi^2^, Reuben M Gibil^28^, Rich-Hope O Abiye-Whyte^29^, Ridhwanullah A Salawu^11^, Ridwan O Maleeq^30^, Ronald A Williams^17^, Saddam Hussain^9^, Sadiq Tijani^20^, Saidu I Alhaji^20^, Samuel B Agaja^32^, Samuel T Oladejo^6^, Sati Samson Bawa^20^, Segun J Oni^6^, Selim S Oriloye^33^, Senyo Gudugbe^24^, Sherif T Maruf^15^, Solomon J Fadamijo^3^, Suleiman T Olorukooba^20^, Taofikat Ikotun^6^, Tathiya N Fakuta^20^, Tavershima M Tsavzua^18^, Temidayo J Alarapon^13^, Temitope J Oyadiran^25^, Timothy K Olagbe^13^, Tobiloba E Oyeyemi^6^, Tochukwu N Enemuo^5^, Tolulope O Ogunrewo^2^, Toluwani O Orungbeja^7^, Udit Agrawal^31^, Umar Mohammed^34^, Uno O Okpo^7^, Vernon Ipomai^14^, Victor Akinkuolie^6^, Yashim I Ignatius^20^, Yau Musa^36^, Yitmwa Ngwan^36^, Yusuf Oladapo Zakariyau^18^

Study design and writing group

Emmanuel O Oladeji, Olorunnisola Olatide, Oluwafisayo Awi, Ridwanullah O Abdullateef, Abdullahi Ringim, Abdulahi Zubair, Imobhio G Okhifun, Adedamola Olaniyi, Oluwatobi Olayode, Noah B Oyedokun, Patrick Okonkwo, Hammed Alabi, Oghofori Obakponovwe, Samuel O Ogunlade.

Data handling and statistical analysis group

Emmanuel O Oladeji, Ridwanullah O Abdullateef, Abdullahi Ringim, Abdulquddus Ajibade, Olorunnisola Olatide, Oluwafisayo Awi, Damilola D Oladeji.

Dissemination and data acquisition group

Abdullahi Ringim, Abdulahi Zubair, Oluwafisayo Awi, Olorunnisola Olatide, Abdul K Bah, Michael K Nzeako, Patrick Okonkwo, Adedamola Olaniyi, Tochukwu Enemuo, Hammed Alabi, Hadayat Ullah, Alex M Kihunyu, Vernon Ipomai, Aniebiet Ubaha, Bismarck C Iwueke, Japheth O Olaku, Cyprian C Nganwuchu, Toluwani O Orungbeja, Adeyemi D Ogunoye, Nura M Aliyu, Saidu I Alhaji, Suleiman T Olorukooba, Taofikat Ikotun, Victor Akinkuolie, Emamizo U Ojo-maliki, Oluwatobi Olayode.

Supervisory Group

Noah B Oyedokun, Oghofori Obakponovwe, Samuel O Ogunlade.

Affiliated institutions

1. Connaught Hospital, Freetown, Sierra Leone.
2. University College Hospital, Ibadan, Nigeria.
3. National Orthopaedic Hospital Igbobi, Lagos, Nigeria.
4. Hayatabad Medical Complex, Peshawar, Pakistan.
5. Ring Road State Hospital, Ibadan, Nigeria.
6. Obafemi Awolowo University Teaching Hospital Complex, Ile-Ife, Nigeria.
7. Lagos State University Teaching Hospital, Lagos, Nigeria
8. Federal Medical Centre, Owo, Nigeria.
9. Ayub Teaching Hospital, Abbottabad, Pakistan.
10. Timergara Hospital, Timergara, Pakistan.
11. University of Ilorin Teaching Hospital, Ilorin, Nigeria.
12. University of Uyo Teaching Hospital, Uyo, Nigeria
13. University of Medical Sciences Teaching Hospital, Ondo, Nigeria.
14. Kenyatta National Hospital, Nairobi, Kenya.
15. National Orthopaedic Hospital, Dala, Nigeria.
16. National Orthopaedic Hospital, Enugu, Nigeria.
17. Komfo Anokye Teaching Hospital, Kumasi, Ghana.
18. Federal Medical Centre Keffi, Nasarawa, Nigeria.
19. University of Maiduguri Teaching Hospital, Maiduguri, Nigeria.
20. Ahmadu Bello University Teaching Hospital, Zaria, Nigeria.
21. State Specialist Hospital, Osogbo, Nigeria.
22. Kyeshero Hospital, Goma, Democratic Republic of Congo.
23. Federal Medical Centre, Bida, Nigeria.
24. Holy Family Hospital, Techiman, Ghana.
25. Primrose Specialist Hospital, Lagos, Nigeria.
26. LAUTECH Teaching Hospital, Ogbomoso, Nigeria.
27. Lagos University Teaching Hospital, Lagos, Nigeria.
28. Korle Bu Teaching Hospital, Accra, Ghana.
29. National Hospital, Abuja, Nigeria.
30. Federal Teaching Hospital, Birnin Kebbi, Nigeria.
31. King George Medical College Hospital, Lucknow, India.
32. Ela Memorial Medical Centre, Ilorin, Nigeria.
33. Uniosun Teaching Hospital, Osogbo, Nigeria.
34. Abubakar Tafawa Balewa University Teaching Hospital, Bauchi, Nigeria.
35. Aminu Kano Teaching Hospital, Kano, Nigeria.
36. Jos University Teaching Hospital, Jos, Nigeria
